# Early-onset torsion dystonia: a novel high-throughput yeast genetic screen for factors modifying protein levels of torsinAΔE

**DOI:** 10.1242/dmm.029926

**Published:** 2017-09-01

**Authors:** Lucía F. Zacchi, John C. Dittmar, Michael J. Mihalevic, Annette M. Shewan, Benjamin L. Schulz, Jeffrey L. Brodsky, Kara A. Bernstein

**Affiliations:** 1 School of Chemistry and Molecular Biosciences, The University of Queensland, St Lucia, QLD 4072, Australia; 2 Department of Biological Sciences, Columbia University, New York, NY 10027, USA; 3 Department of Microbiology and Molecular Genetics, University of Pittsburgh School of Medicine, 5117 Centre Avenue, UPCI Research Pavilion, 2.42e, Pittsburgh, PA 15213, USA; 4 Department of Biological Sciences, A320 Langley Hall, University of Pittsburgh, Pittsburgh, PA 15260, USA

**Keywords:** Yeast, Genetic screen, Protein levels, Protein disulfide isomerase, TorsinA, Dystonia

## Abstract

Dystonia is the third most common movement disorder, but its diagnosis and treatment remain challenging. One of the most severe types of dystonia is early-onset torsion dystonia (EOTD). The best studied and validated EOTD-associated mutation, torsinAΔE, is a deletion of a C-terminal glutamate residue in the AAA+ ATPase torsinA. TorsinA appears to be an endoplasmic reticulum (ER)/nuclear envelope chaperone with multiple roles in the secretory pathway and in determining subcellular architecture. Many functions are disabled in the torsinAΔE variant, and torsinAΔE is also less stable than wild-type torsinA and is a substrate for ER-associated degradation. Nevertheless, the molecular factors involved in the biogenesis and degradation of torsinA and torsinAΔE have not been fully explored. To identify conserved cellular factors that can alter torsinAΔE protein levels, we designed a new high-throughput, automated, genome-wide screen utilizing our validated *Saccharomyces cerevisiae* torsinA expression system. By analyzing the yeast non-essential gene deletion collection, we identified 365 deletion strains with altered torsinAΔE steady-state levels. One notable hit was *EUG1*, which encodes a member of the protein disulfide isomerase family (PDIs). PDIs reside in the ER and catalyze the formation of disulfide bonds, mediate protein quality control and aid in nascent protein folding. We validated the role of select human PDIs in torsinA biogenesis in mammalian cells and found that overexpression of PDIs reduced the levels of torsinA and torsinAΔE. Together, our data report the first genome-wide screen to identify cellular factors that alter expression levels of the EOTD-associated protein torsinAΔE. More generally, the identified hits help in dissecting the cellular machinery involved in folding and degrading a torsinA variant, and constitute potential therapeutic factors for EOTD. This screen can also be readily adapted to identify factors impacting the levels of any protein of interest, considerably expanding the applicability of yeast in both basic and applied research.

## Abstract (precis)

**Summary:** A novel high-throughput, automated, genome-wide yeast screen was used to identify genetic modifiers of the steady-state levels of proteins of the dystonia-associated torsinAΔE variant. This article has an associated First Person interview with the first author of the paper as part of the supplementary information.

## INTRODUCTION

Dystonia is a movement disorder characterized by sustained involuntary muscle contractions leading to abnormal, often repetitive movements and/or postures (Albanese et al., 2013). One of the most severe types of dystonia is early-onset torsion dystonia (EOTD) (Bragg et al., 2011; Bruggemann and Klein, 2010). The severity of EOTD is due to the early age of onset (∼12 years, with the majority of the cases beginning before age 26) and the potential to compromise all limbs in the body (Ozelius and Lubarr, 2014; O'Riordan et al., 2004). The best studied EOTD mutation is the deletion of a GAG codon in the *DYT1* gene, which eliminates a glutamate residue (ΔE) at position 302/303 in the protein torsinA (torsinAΔE) (Klein et al., 1998; Ozelius et al., 1998, 1997, 1992; Kramer et al., 1994). Although torsinAΔE is encoded by a dominant allele and appears to display a dominant-negative phenotype (Torres et al., 2004; Hewett et al., 2008; Bressman et al., 1989), only ∼30% of heterozygous carriers develop dystonia, indicating that additional factors contribute to EOTD development (Bressman, 2007). These additional factors may directly regulate torsinA or torsinAΔE expression or function, or they may indirectly impact disease by regulating other pathways required for disease onset.

The cellular role of torsinA is not completely understood. TorsinA has been implicated in lipid metabolism and in the modification of cellular/nuclear envelope (NE) architecture (Grillet et al., 2016; Kamm et al., 2004; Goodchild et al., 2005; Tanabe et al., 2016), and it may function as a chaperone associated with protein quality control and protein degradation (Nery et al., 2011; Burdette et al., 2010; Chen et al., 2010; Thompson et al., 2014). Indeed, torsinA function impacts the degradation and trafficking of membrane proteins and influences synaptic vesicle recycling and dopamine neurotransmission (Torres et al., 2004; Nery et al., 2011; Balcioglu et al., 2007; Granata et al., 2008; Zhao et al., 2008; O'Farrell et al., 2009; Warner et al., 2010; Hewett et al., 2008; Liang et al., 2014). TorsinAΔE is defective for these processes (Bragg et al., 2011; Granata and Warner, 2010). Therefore, EOTD may be linked to torsinAΔE-dependent defects in protein homeostasis.

TorsinA is an unusual member of the AAA+ ATPase family of chaperone-like proteins (Ozelius et al., 1998; Hanson and Whiteheart, 2005; Rose et al., 2015). Some of the features that make this AAA+ ATPase unique include its residence in the endoplasmic reticulum (ER) lumen (Liu et al., 2003; Bragg et al., 2004), that it is a glycoprotein with intramolecular disulfide bonds (Bragg et al., 2004; Zhu et al., 2008, 2010) and that it assembles into a heterohexamer, which is required for ATPase activity (Zhao et al., 2013; Rose et al., 2015; Brown et al., 2014; Sosa et al., 2014). In addition, torsinA is a monotopic protein that associates with the ER membrane through an N-terminal hydrophobic domain that retains torsinA in the ER and is required for hexamer formation (Liu et al., 2003; Callan et al., 2007; Vander Heyden et al., 2011; Li et al., 2014). Therefore, during biogenesis, torsinA acquires numerous post-translational modifications in the ER that require distinct enzymes: the oligosaccharyltransferase for *N*-linked glycosylation, the protein disulfide isomerases (PDIs) for disulfide bond formation, and the chaperone/lectin network for folding and assembly (Zacchi et al., 2016, 2014). Interactions with these ER-resident machineries are crucial because the ΔE mutation decreases torsinA stability and targets torsinAΔE for degradation through a different pathway than wild-type torsinA (Giles et al., 2008; Gordon and Gonzalez-Alegre, 2008). Nevertheless, our understanding of the cellular factors that facilitate torsinA biogenesis is incomplete.

To uncover new factors that impact folding and degradation of torsinA and torsinAΔE, we designed a new high-throughput, automated, genetic screen in *Saccharomyces cerevisiae*. Yeast is an ideal system in which to investigate fundamental questions on protein biogenesis and secretory-pathway function because these processes are highly conserved from yeast to humans (Botstein and Fink, 2011; Winderickx et al., 2008). Yeast is also an excellent model for genetic and biochemical studies of proteins involved in human diseases and for identifying therapeutic targets for disease (Khurana et al., 2015; Sarto-Jackson and Tomaska, 2016; Tenreiro and Outeiro, 2010; Ju et al., 2011; Walberg, 2000; Kolb et al., 2011; Gelling and Brodsky, 2010). Yeast has also been extensively used to perform genome-wide analyses, providing key insights into the etiology of multiple neurological diseases (Willingham et al., 2003; Ju et al., 2011; Miller-Fleming et al., 2008; Tyedmers et al., 2008; Knight et al., 1999). Therefore, we took advantage of our previously validated torsinA/ΔE yeast heterologous expression system (Zacchi et al., 2014) and performed an unbiased genome-wide screen to analyze torsinAΔE levels when expressed in the yeast non-essential gene deletion collection. We identified 365 gene deletions that led to alterations in torsinAΔE protein levels in yeast. One hit that stood out was *EUG1*, which encodes a PDI. PDIs are important for the folding and quality control of proteins with disulfide bonds (Okumura et al., 2015). PDIs are associated with multiple diseases, including neurological diseases, and are a target of therapeutic development (Ellgaard and Ruddock, 2005; Atkin et al., 2006; Walker et al., 2010; Chen et al., 2013; Xu et al., 2014; Ali Khan and Mutus, 2014; Perri et al., 2015; Parakh and Atkin, 2015; Liu et al., 2015; Kaplan et al., 2015; Torres et al., 2015). To support these data, we confirmed that the expression of select mammalian PDIs reduced the levels of torsinA and torsinAΔE in human cells. Detailed exploration of additional hits from our screen may help us to understand how torsinA folds and assembles, how the torsinAΔE variant and wild-type torsinA are differentially degraded, and whether dedicated chaperones – some of which are being targeted pharmacologically (Brandvold and Morimoto, 2015; Brodsky and Chiosis, 2006; Balch et al., 2008) – impact torsinAΔE penetrance.

## RESULTS

### A yeast genetic screen to identify modifiers of torsinAΔE stability

Most yeast genetic screens rely on the measurement of colony growth. However, expression of heterologous proteins does not always lead to an overt growth phenotype. Indeed, expression of torsinA or torsinAΔE in yeast has no effect on growth irrespective of the strain background or even under stress conditions (Zacchi et al., 2014; Valastyan and Lindquist, 2011). Thus, to identify genes or pathways that alter torsinAΔE expression levels, we designed a new method for the high-throughput analysis of heterologously expressed proteins in the *S. cerevisiae* deletion collection library (see Materials and methods for details).

**Fig. 1.**
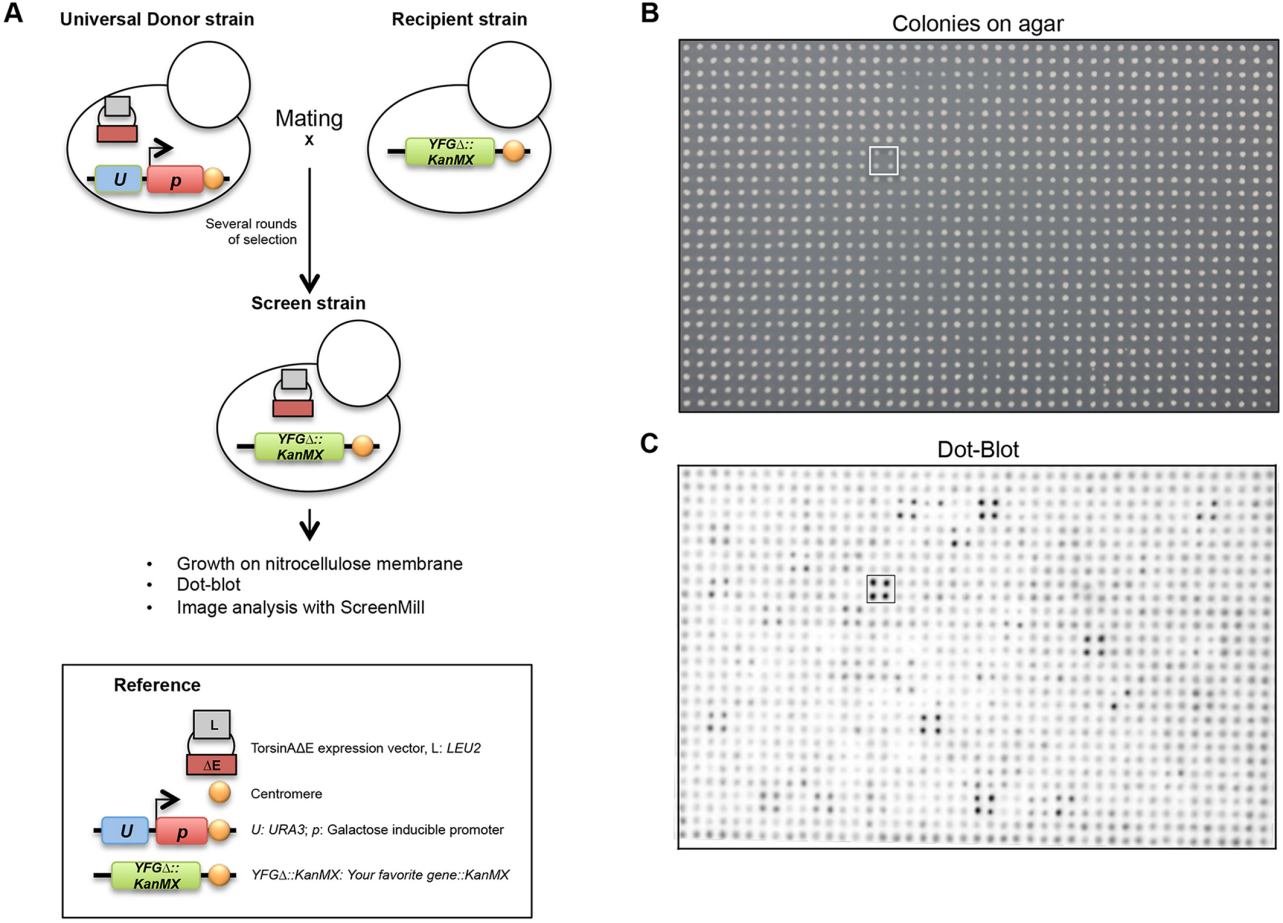
**Schematic representation of the genetic screen for genes affecting torsinAΔE expression levels.** (A) For the high-throughput transfer of the expression vector for torsinAΔE-HA, a universal donor strain genetically engineered such that all its chromosomes contain a *URA3* gene (*U*, blue box) and a galactose-inducible promoter (*p*, red box) neighboring the centromeres (Reid et al., 2011) was transformed with a LEU2-marked (L, gray box) expression vector for torsinAΔE-HA (ΔE, maroon box). This donor strain was mated in quadruplicate to the yeast non-essential gene deletion collection library [each strain carries the deletion of one gene: Your favorite gene (*yfg*)Δ::*KanMX*, green box]. After several rounds of selection, the screening strains were generated. Each screening strain contains the haploid genomic content (with a unique gene deletion) of each deletion collection strain, the expression vector for torsinAΔE-HA and no chromosomes from the donor strain. Using this technique, we examined torsinAΔE-HA expression in ∼90% of the deletion collection. See details in the Materials and methods and Results sections. (B) Example of an array of screened colonies on selective medium, and (C) the corresponding dot-blot image. The white and black squares indicate a strain spotted in quadruplicate [*rsb1*Δ, 3.12× higher normalized signal (Table S1)]. The border strains are not shown in this image.

To efficiently express torsinAΔE in yeast, we employed a high-copy expression plasmid for constitutive expression of C-terminally hemagglutinin (HA)-tagged torsinAΔE (pRS425-GPD-torsinAΔE-HA) (Zacchi et al., 2014). The C-terminal HA tag does not impact torsinA or torsinAΔE localization, function or stability in mammalian cells, and does not affect growth relative to wild-type yeast cells expressing an empty vector or untagged torsinA (Zacchi et al., 2014; Torres et al., 2004; Naismith et al., 2004, 2009; Giles et al., 2008; Valastyan and Lindquist, 2011). Next, we took advantage of selective ploidy ablation (Reid et al., 2011), a technique which allows transfer of the pRS425-GPD-torsinAΔE-HA plasmid into the yeast deletion collection through a simple mating procedure. In this method, a donor strain (Table 1, strain W8164-2C) is first transformed with the pRS425-GPD-torsinAΔE-HA plasmid. The donor strain contains a galactose-inducible promoter and a counterselectable *URA3* gene in close proximity to the centromere in each of its 16 chromosomes (Fig. 1A) (Reid et al., 2011). The vector-containing donor strain is then mated to the deletion library collection. Haploid cells containing only the deletion library chromosomes and the pRS425-GPD-torsinAΔE-HA plasmid are generated by pinning the ‘diploid’ yeast onto medium supplemented with 5-fluoroorotic acid (5-FOA; which counter-selects for the *URA3*-expressing chromosomes) and galactose (which destabilizes the chromosomes at the centromere). These growth conditions cause selective loss of heterozygosity during mitotic growth, generating haploid strains lacking the donor chromosomes but maintaining the vector of interest and the unique gene deletion of the original strain (Reid et al., 2011).

**Table 1.**
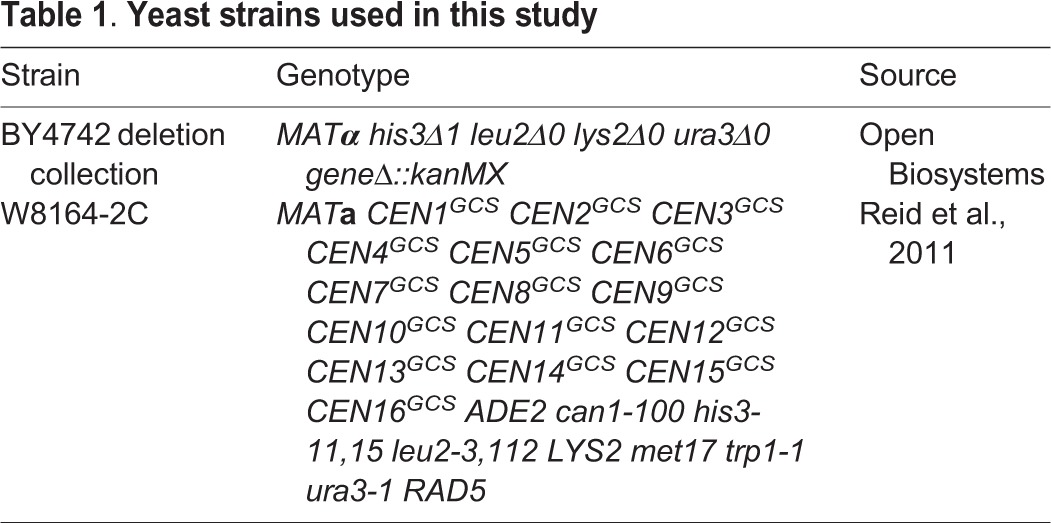
**Yeast strains used in this study**

Using a robotic pinner, we performed each mating independently and in quadruplicate (Fig. 1B; as an example, the white square indicates the colonies associated with one screening hit, the *rsb1*Δ deletion strain, mated in quadruplicate). Because colonies on the edges of the plates exhibit a growth advantage, we utilized a library containing a *his3*Δ strain along the plate perimeter (Fig. S1A). To determine how individual gene deletions impacted the steady-state levels of torsinAΔE-HA, each strain was replica-pinned on top of nitrocellulose membranes placed on selective medium (Gelling et al., 2012). The yeasts were incubated for 8 h at 30°C on the membrane and lysed *in situ*, thus enabling the total cellular protein pool to adhere to the nitrocellulose membrane (Gelling et al., 2012). After washing the cellular debris from the membrane surface, the membranes were prepared for western (dot) blotting (Fig. 1C). For example, in Fig. 1C, the black square shows the dot-blot signal of the *rsb1*Δ strain pinned in quadruplicate. The images were then analyzed using a modified version of ScreenMill (Dittmar et al., 2010) adapted to measure the dot-blot signal (see Materials and methods and Appendix S1). To identify strains with different torsinAΔE expression levels, we normalized the signal for each strain to the average signal of the eight strains immediately surrounding each colony of interest (Dittmar et al., 2010) (Fig. S1B). This technique is based on the assumption that the majority of the deletion strains will not display altered torsinAΔE-HA expression levels. The comparison of the signal of each strain to the signal of the eight immediately bordering strains was designed to counterbalance occasional artefacts during development of the dot blot that caused differences in the background signal across areas of each membrane. An important advantage of the dot-blot technique compared to other protein quantitative methods, such as fluorescence microscopy, which suffers from interfering yeast autofluorescence (Mazumder et al., 2013), is that highly selective antibodies specifically detect the expressed protein. Finally, we selected those strains whose normalized signal intensity was significantly different than the average (210 strains, *P*<0.1; Table S1). We also manually included hits from plates that could not be assessed by the script and false negatives identified by visual inspection of the images. Ultimately, we obtained a list of 365 genes that alter the steady-state level of torsinAΔE-HA (Table S2).

### Gene ontology analysis of hits from the genetic screen and hit selection

**Fig. 2.**
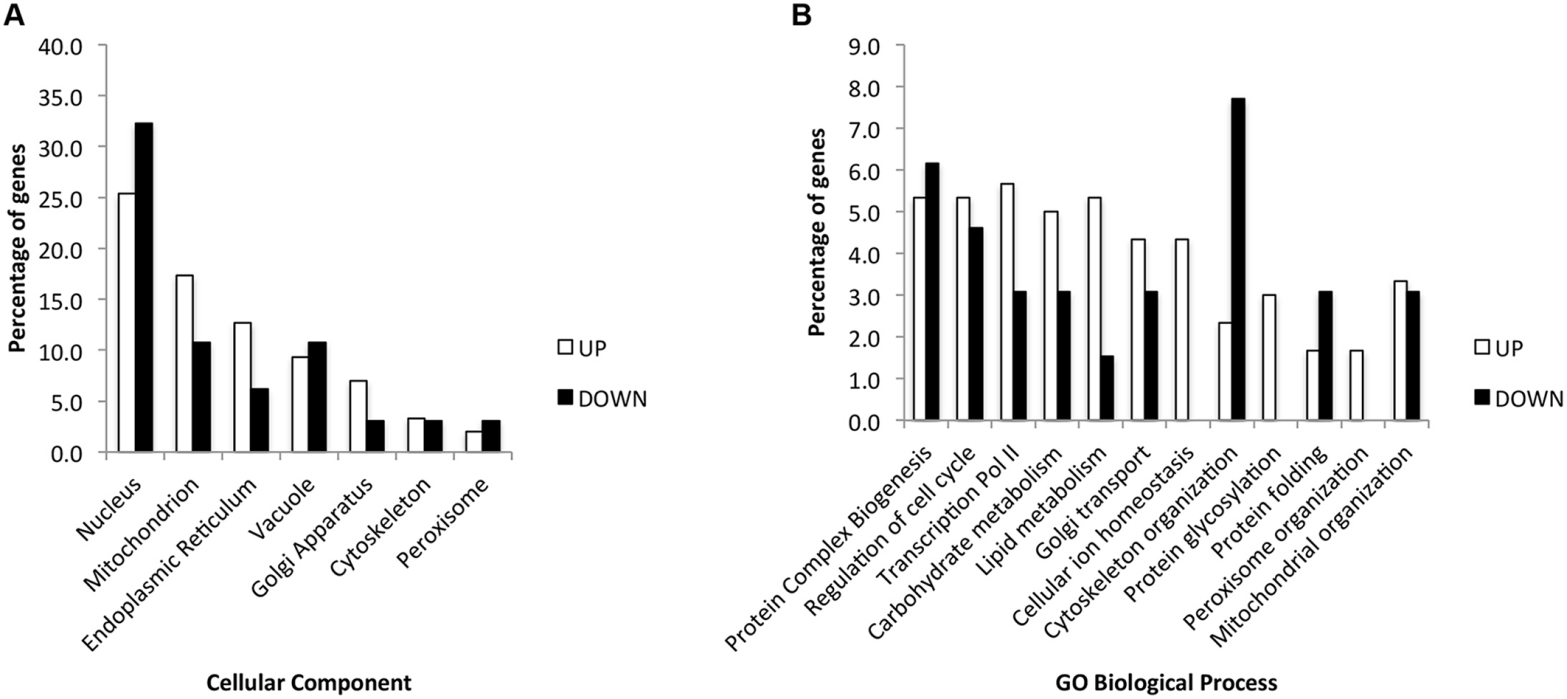
**Distribution of hits in categories by gene ontology (GO).** GO-term analysis of 365 yeast genes obtained as hits from the screen selecting for: (A) component and (B) biological process. Graphs display the percentage of hits associated with the different categories that showed higher normalized levels of torsinAΔE (UP, white bars) or lower normalized levels (DOWN, black bars). Only a selection of the categories are presented in the graphs. For full details of the analysis, please refer to Table S3.

We next grouped the hits to identify significant gene ontology (GO) categories (Fig. 2; Table S3). The majority of the hits were in genes associated with the nucleus (97 genes, 26.6%), the ER and Golgi (65 genes, 17.8%), the mitochondria and peroxisome (67 genes, 18.3%), the vacuole (35 genes, 9.6%) and the cytoskeleton (12 genes, 3.3%) (Fig. 2A; Table S3, Component). Of these, genes associated with the secretory pathway were significantly represented (lumenal proteins, *P*<0.002 and Golgi, *P*<0.005, Table S3, GO). The hits were associated with a wide variety of cellular processes, including, among others: RNA polymerase II transcription (19 genes, 5.2%); protein folding, glycosylation and complex biogenesis (36 genes, 9.9%); lipid metabolism (17 genes, 4.7%); and cellular ion homeostasis (13 genes, 3.6%) (Fig. 2B; Table S3, Process). The categories ‘Cellular ion homeostasis’, ‘Protein glycosylation’ and ‘Peroxisome organization’ were only associated with hits that led to higher steady-state levels of torsinAΔE-HA (Fig. 2B; Table S3, Process). In particular, the GO categories of ‘*N*-linked glycosylation’ and ‘Cellular iron ion homeostasis’ were significantly represented (*P*<0.015 and *P*<0.023, respectively; Table S3, GO), as well as ‘ATP binding’ (35 genes, *P*<0.021). Very few GO categories were significantly represented in the hits leading to lower steady-state levels of torsinAΔE-HA (*P*<0.05; Table S3, GO). These GO categories included ‘Purine metabolism’ (2 genes) and ‘Nucleus’ (24 genes).

To select hits for validation, we applied the following stringent criteria. First, we focused on yeast genes from Table S2 for which there were human homologs (Table S4). Second, we identified those human homologs that are linked to human diseases (Table S5), since a protein associated with other diseases has a higher chance of being targeted for therapeutic development. Third, we identified which human homologs were expressed at the mRNA or protein level in the brain (Table S5). Finally, because torsinA is a neurological disease of the central nervous system (Bragg et al., 2011), we identified those human genes associated with neurological diseases (Table 2). In this way, we first generated a list of 656 human homologs corresponding to the yeast hits (Table S4). The number of human homologs is larger than the number of hits because the mammalian genome is more complex and redundant than the yeast genome. Of these 656 human genes, 141 were associated with 182 human pathologies (Table S5). Most of the genes were expressed in the brain (122 out of 141, Table S5, in bold). Finally, of these 122 potential genes of interest, 33 were associated with a range of neurological diseases, including Parkinson's, Alzheimer's, rapid onset dystonia parkinsonism, and deafness, dystonia and central hypomyelination, among others (Table 2).

**Table 2.**
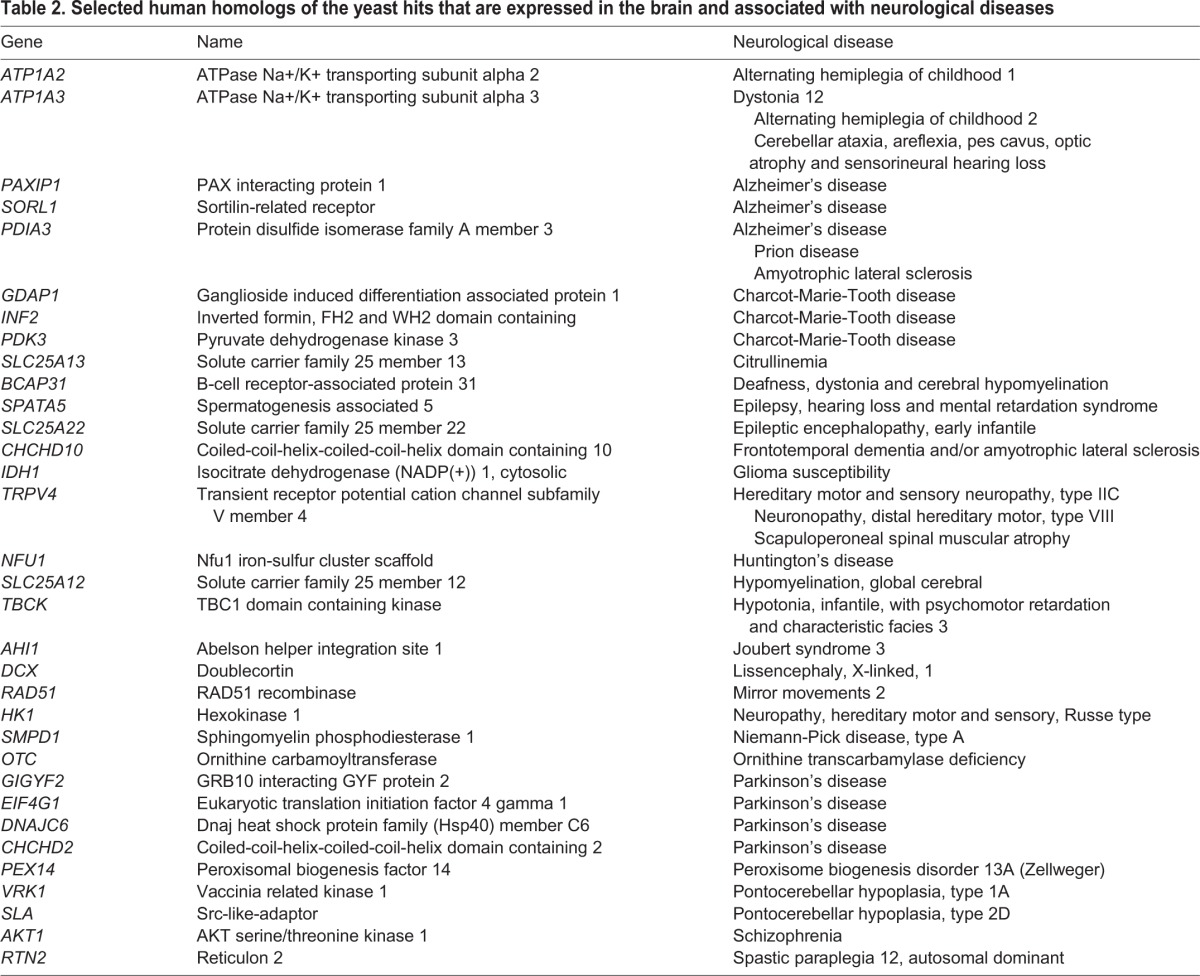
**Selected human homologs of the yeast hits that are expressed in the brain and associated with neurological diseases**

### Overexpression of mammalian protein disulfide isomerases lowers the levels of torsinA and torsinAΔE

Through the analysis outlined above, we generated a final list of 33 genes (Table 2). Based on torsinA function and residence, one notable hit was *EUG1*, which encodes a PDI family member. PDIs form, reduce and isomerize disulfide bonds in the ER (Vembar and Brodsky, 2008; Hatahet and Ruddock, 2009). Disulfide bond formation is critical for the correct folding and function of a large portion of the secretory proteome, including torsinA (Hatahet and Ruddock, 2009; Zhu et al., 2010). TorsinA and torsinAΔE contain six conserved cysteines (Cys44, Cys49, Cys50, Cys162, Cys280 and Cys319) (Zhu et al., 2008). Two N-terminal cysteines (Cys49, Cys50) mark the site for a proteolytic cleavage that removes the hydrophobic N-terminus (Zhao et al., 2016), whereas the C-terminal cysteines (Cys280 and Cys319) form an intramolecular disulfide bond (Zhu et al., 2008). The remaining two cysteines (Cys44 and Cys162) have been proposed to form an additional intramolecular disulfide bond (Zhu et al., 2008). Cys319 is located within the non-canonical nucleotide-interacting sensor-II motif, and nucleotide binding to sensor-II depends on the redox status of Cys319 (Zhu et al., 2010). The ΔE mutation disrupts the redox-sensing ability of this region, impairing torsinA's interaction with LAP1B and LULL1 (Zhu et al., 2010). Thus, cysteines in torsinA maintain the protein's conformation and function, supporting a role for the PDIs in torsinA biogenesis and/or function.

Some PDIs act as chaperones, recognizing hydrophobic regions within nascent proteins and shielding them to prevent protein aggregation (Fink, 1999; Vembar and Brodsky, 2008; Buck et al., 2007; Song and Wang, 1995; Kimura et al., 2004; Ellgaard and Ruddock, 2005). PDIs also facilitate the degradation of some substrates (Gillece et al., 1999; Wang and Chang, 2003; Gauss et al., 2011; Grubb et al., 2012) and are involved in a myriad of human diseases, including cancer, diabetes, infectious diseases, and neurological diseases including prion and Alzheimer's diseases, cerebral ischemia and amyotrophic lateral sclerosis (Ali Khan and Mutus, 2014; Torres et al., 2015; Jeon et al., 2014; Chen et al., 2013; Liu et al., 2015; Parakh and Atkin, 2015). Consequently, small-molecule modulators of PDIs are under development (Parakh and Atkin, 2015; Xu et al., 2014, 2012). Considering our selection criteria, we chose to study how select members of the highly conserved group of PDIs affect torsinA and torsinAΔE levels in human cells.

There are >20 members of the PDI family encoded in the human genome (Okumura et al., 2015). To identify the closest human homolog of Eug1, we performed a BLASTP alignment and found that ERp57 (28% identity, 176 bits, 6e-48) was most similar. ERp57 also has a similar domain organization as two other mammalian PDIs, PDI and ERp72. ERp57 and PDI contain two catalytically active thioredoxin-like domains (required for disulfide bond formation or isomerization) that flank two central redox-inactive thioredoxin-like domains that recognize protein-folding intermediates; in contrast, ERp72 contains three catalytically active domains and two inactive domains (Okumura et al., 2015). These PDIs have both enzymatic and chaperone activity. ERp57, PDI and ERp72 are also among the best-studied PDIs and are expressed in multiple tissues, including the brain (Uhlen et al., 2015). Therefore, we selected ERp57, PDI and ERp72 to validate our screen results and to test whether members of the PDI family impact the steady-state levels of torsinA and torsinAΔE.

**Fig. 3.**
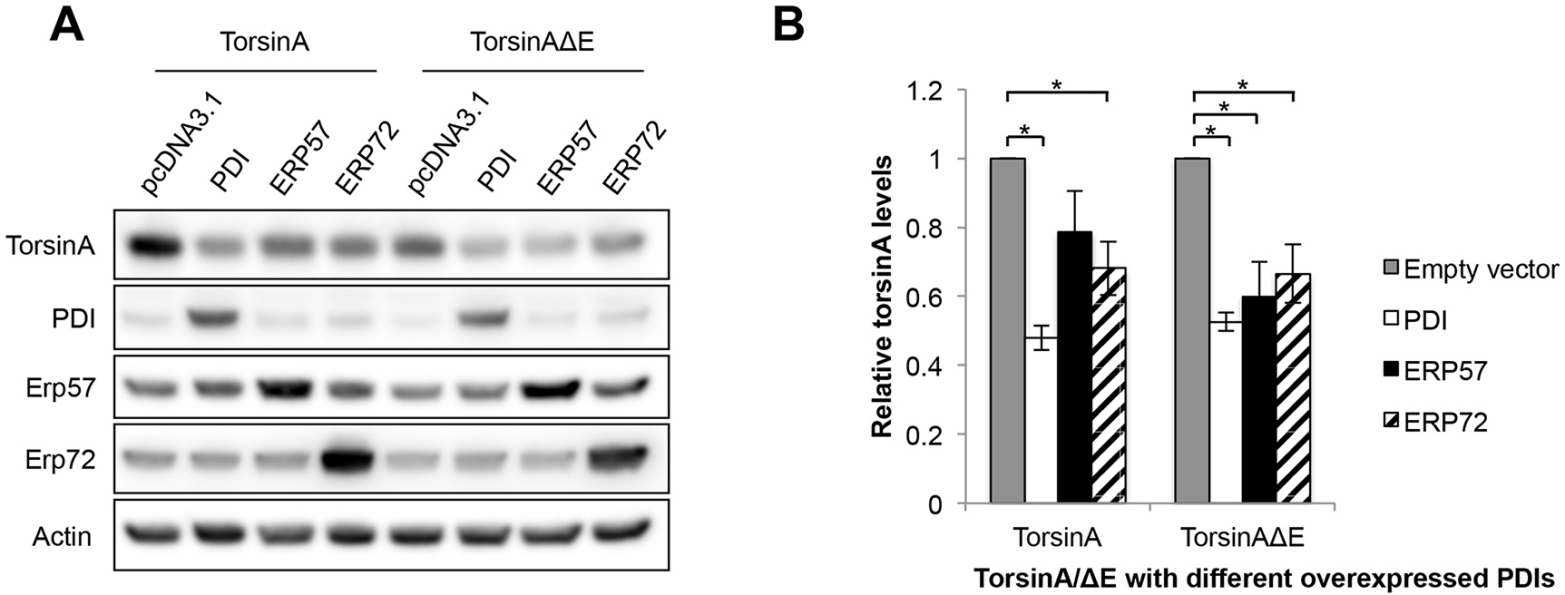
**Overexpression of mammalian protein disulfide** **isomerases decreases the steady-state expression levels of torsinA and torsinAΔE.** (A) TorsinA and torsinAΔE were co-expressed in HeLa cells with PDI, ERp57 or ERp72, or with an empty vector. A total of 24 h after transfection the cells were harvested, and protein extracts were resolved by SDS-PAGE and examined by western blot analysis. Protein levels were measured using ImageJ. (B) The graph shows the means±s.e.m. of the normalized levels of torsinA and torsinAΔE when co-expressed with a particular PDI relative to co-expression with an empty vector control. Data were obtained from three independent experiments with at least one replica/experiment (total *n*=5). *P<0.05 for the following comparisons: co-expression of torsinA/torsinAΔE and PDI, torsinAΔE and ERp57, or torsinA/torsinAΔE and ERp72 vs torsinA/torsinAΔE and the empty vector control.

In our yeast screen, deletion of *EUG1* increased torsinAΔE levels ∼1.6-fold (Table S1). We reasoned that overexpression of select PDIs in mammalian cells may reduce torsinAΔE levels. To this end, we co-expressed torsinA or torsinAΔE with vectors engineered for the expression of PDI, Erp72 or Erp57, or with an empty vector control (Fig. 3). We found that elevated levels of PDI and ERp72 significantly decreased the steady-state expression of both torsinA and torsinAΔE (Fig. 3): 48% of torsinA and 53% of torsinAΔE remained when PDI was co-overexpressed compared to co-transfection with an empty vector, and 68% of torsinA and 66% of torsinAΔE remained when ERp72 was co-overexpressed compared to co-transfection with an empty vector (*P*<0.05). ERp57 overexpression also reduced both torsinA and torsinAΔE levels, but the difference in expression levels was only significant for torsinAΔE (Fig. 3): 79% of torsinA and 60% of torsinAΔE remained when ERp57 was co-overexpressed compared to co-transfection with empty vector (*P*<0.05 for torsinAΔE). Co-overexpression of an unrelated ER chaperone, Grp170, which was not a hit in the screen, had no effect on the levels of torsinA compared to a vector control, indicating that the effect observed for the PDIs is not an artefact of the co-overexpression of an ER lumenal chaperone (Fig. S2). In summary, this novel genetic screen, which represents the first genome-wide analysis for factors that affect torsinAΔE expression, has produced a number of hits that may similarly affect torsinAΔE expression in humans and will help us understand the cellular machinery involved in torsinA and torsinAΔE biogenesis and degradation.

## DISCUSSION

Since the identification of torsinAΔE two decades ago (Kramer et al., 1994; Ozelius et al., 1997), considerable progress has been achieved to understand the cellular function and structure of torsinA and the molecular consequences of the ΔE mutation (Demircioglu et al., 2016; Zhu et al., 2010; Brown et al., 2014; Rose et al., 2015). Unfortunately, the precise function of torsinA, the impact of the ΔE mutation on torsinA and in cellular function, and a complete understanding of the cellular pathophysiology leading to disease are still lacking. However, the molecular characteristics of torsinA together with torsinAΔE's diminished stability and altered degradation pathway compared to wild-type torsinA (Gordon and Gonzalez-Alegre, 2008; Giles et al., 2008) indicate that it should be possible to identify cellular factors that impact torsinA and torsinAΔE biogenesis or degradation, and that some of these factors may be different for torsinA and torsinAΔE. We hypothesized that these factors are conserved, and we designed a new and unbiased genome-wide genetic screen in the model eukaryote, the yeast *S. cerevisiae*, to identify them.

**Fig. 4.**
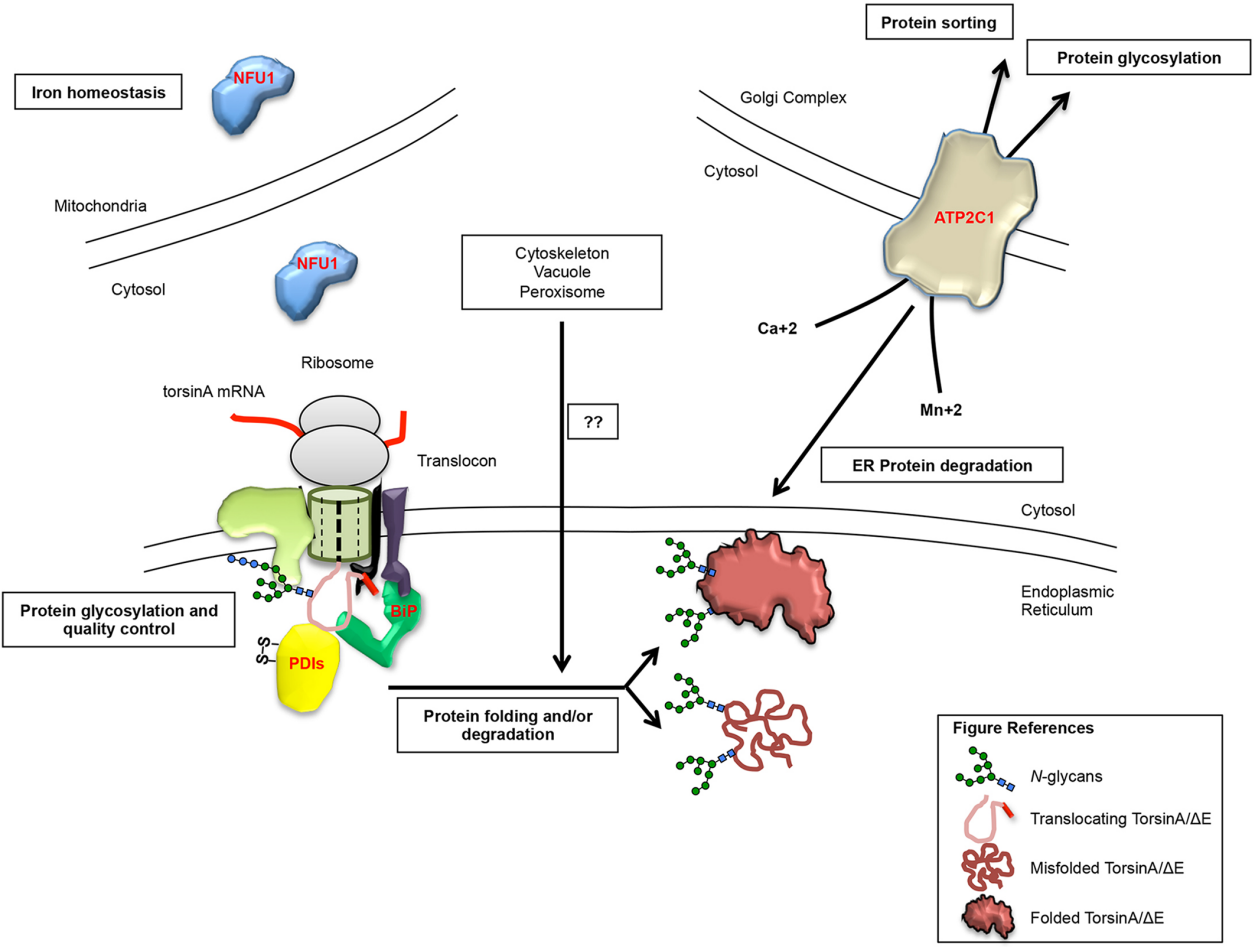
**Summary of the physiological roles of select screen hits.** As torsinA and torsinAΔE are translocated into the ER they are bound and modified by a number of enzymes/chaperones, which ‘decide’ on their folding or degradation fate. Some of the relevant hits mentioned in the Results and Discussion sections are marked in red, including PDIs, NFU1 and ATP2C1, as well as the Hsp70 BiP, which we previously validated as a pro-folding factor for both torsinA and torsinAΔE (Zacchi et al., 2014). In this screen, we identified conserved genes involved in ER/Golgi protein glycosylation, ion homeostasis, and ER protein folding and/or degradation, among others, as factors impacting torsinAΔE levels (see Tables S2 and S3). The screening hits identified were associated with multiple cellular compartments, including the ER, Golgi complex, mitochondria, vacuole and peroxisome, as well as the cytoskeleton. Identified hits may directly or indirectly alter the steady-state levels of torsinAΔE, and may uncover novel links between other processes and torsinAΔE biogenesis and degradation.

We took advantage of our validated yeast heterologous expression system for torsinA (Zacchi et al., 2014) and our expertise in the development of yeast genetic screens (Gelling et al., 2012; Dittmar et al., 2010; Reid et al., 2011) to develop a new workflow to identify genetic modifiers of torsinAΔE biogenesis (Figs 1 and 4; Fig. S1). We analyzed the effect of ∼90% of the non-essential *S. cerevisiae* genes on torsinAΔE cellular protein levels (Fig. 1) and identified 365 hits involved in a variety of cellular processes (Fig. 2; Tables S2 and S3). We then searched for human homologs of the yeast genes identified in the screen, and prioritized hits that fulfilled the criteria of being expressed in the brain and associated with a neurological disease (33 candidates selected, Table 2). The final criterion for selection required that we could predict an interaction between the hit and torsinA based on the known function of the protein encoded by the identified hit and its residence in the ER. Thus, we focused on *EUG1*, which encodes a PDI. As noted above in the Introduction and Results sections, PDIs are associated with human diseases, including neurological diseases (Walker et al., 2010; Perri et al., 2015), and PDI modulators for therapeutic treatment are being developed (Ali Khan and Mutus, 2014; Xu et al., 2014; Okumura et al., 2015; Torres et al., 2015; Kaplan et al., 2015). Moreover, disulfide bonds play an important role in torsinA function (Zhu et al., 2008, 2010). Interestingly, each of the three human PDIs most closely related to yeast Eug1 lowered torsinA and torsinAΔE steady-state levels (Fig. 3). Because a pro-degradative role for PDIs has been described for several substrates (Gillece et al., 1999; Wang and Chang, 2003; Gauss et al., 2011; Grubb et al., 2012), we suggest that torsinA and torsinAΔE are also PDI substrates.

Our screen has uncovered a large number of additional factors potentially involved in torsinAΔE biogenesis and/or degradation (Fig. 4). One of the GO categories that was significantly represented was associated with glycoprotein biosynthesis (Tables S2 and S3). Protein glycosylation, the addition of sugar moieties to proteins through *N*- or *O*-linkages, is one of the most important and versatile post-translational modifications (Zacchi and Schulz, 2016). Glycans are critical for the development, function and homeostasis of the nervous system (Freeze et al., 2015), and many of the over 100 congenital disorders of glycosylation (CDGs) are accompanied by neurological defects (Freeze et al., 2015). Importantly, the carrier frequency of alleles associated with CDGs in the general population is unusually high (∼1/1000 individuals in the USA) (Freeze et al., 2014) and protein glycosylation is a current target of pharmacological therapy (Dalziel et al., 2014). Further, torsinA is a glycoprotein, with two sites for *N*-linked glycosylation that are required for proper subcellular localization and stability (Zacchi et al., 2014; Bragg et al., 2004). Thus, mutations in genes associated with glycosylation could be risk factors in EOTD onset.

Another significant GO category identified in the screen was ‘Cellular iron ion homeostasis’ (*P*<0.023) (Fig. 4; Table S3, GO). Iron accumulation in the brain has been observed in multiple neurological disorders, and iron chelators are used to treat Parkinson's disease and Friedrich's ataxia (Rouault, 2013; Schneider et al., 2012). One of the hits in this GO category was *NFU1* (Table 2; Table S2). TorsinAΔE levels were 2.7-fold higher in the *nfu1*Δ strain (Table S1). Human NFU1 is associated with mitochondrial disorders that may present neurological symptoms (Papsdorf et al., 2015; Nizon et al., 2014; Navarro-Sastre et al., 2011). NFU1 has also been recently implicated in Huntington's disease, supporting an association between iron homeostasis and proteostasis in neurological disorders (Papsdorf et al., 2015; Mancuso et al., 2007). Because EOTD appears to be a proteostasis disorder (Liang et al., 2014; Nery et al., 2011), and genes associated with metal-induced diseases are being considered as therapeutic targets (Flynn et al., 1991; Wong et al., 1999; Lodi et al., 2006; Schneider et al., 2012; Rouault, 2013; Jomova and Valko, 2011), NFU1 is another EOTD modifier candidate to be studied in future efforts.

The last GO category that was significantly represented was ‘ATP binding’. One intriguing candidate for future validation is *PMR1*, or ATP2C1 in humans (Fig. 4; Table S2). ATP2C1 is a Ca^2+^ and Mn^2+^ pump localized in the Golgi complex and is associated with Hailey-Hailey disease and other disorders (Hu et al., 2000; Vanoevelen et al., 2005; Dang and Rao, 2016). Complementation studies of the yeast *pmr1*Δ with human ATP2C1 demonstrated that ATP2C1 is a functional ortholog of *PMR1* (Ton et al., 2002). In our screen, the levels of torsinAΔE were 2.0-fold higher in the *pmr1*Δ strain (Table S1), which was verified by western blot (data not shown). ATP2C1 has a high affinity for Ca^2+^ (Vanoevelen et al., 2005). Defects in Pmr1 and ATP2C1 sensitize cells to ER stress and lead to Ca^2+^-dependent defects in protein cargo sorting and in Mn^2+^-dependent defects in Golgi protein glycosylation and ER protein degradation (Ramos-Castaneda et al., 2005; Durr et al., 1998). Cellular Ca^2+^ homeostasis has been increasingly linked to dystonia, both through the analysis of multiple dystonia-associated genes (Charlesworth et al., 2012; Domingo et al., 2016) and from mouse models suggesting a role for Ca^2+^ dysregulation in the pathogenesis of DYT1 dystonia (Beauvais et al., 2016). Therefore, ATP2C1 is another candidate for further exploration.

More than 300 additional hits that alter torsinAΔE steady-state protein levels were also identified (Fig. 4). Many of these genes are directly associated with ER protein translocation, folding and degradation (*DER1*, *SOP4*, *YET2*, *SEC72* and *ERV2*). Other ER-associated genes are involved in different processes, including ER morphology (*RTN2*, *PER33*), lipid biosynthesis (*NSG1*, *AYR1*, *LAC1*, *SAC1*, *ERG6*, *RSB1*), protein trafficking (*YCK1*, *SVP26*, *SOP4*, *SAC1*, *ERV41*), ion transport (*YKE4*, *ZRT2*), complex assembly (*VPH2*) and mRNA tethering (*SHE2*). Similarly, we identified more than 100 hits associated with the Golgi complex, mitochondria, cytoskeleton, vacuole and/or peroxisome (Fig. 4). These genes may uncover novel connections between torsinA, the ER and other organelles.

Finally, we noted that 87 genes (23.8% of the hits from Table S2) encoded proteins with putative or unknown functions or dubious open reading frames (ORFs). Many of the dubious ORFs overlap with known genes and represent insertions in verified ORFs, likely affecting their expression/function (Table S6). In some cases, the interrupted ORF was functionally related to other genes that were hits in the screen. For example, *YGL137W* overlaps with *LSB1*, and *LSB1*'s paralog *PIN3* and the functionally related *LSB3* were identified as hits (Tables S2 and S6). The remaining genes that were not associated with a known function but that impact the expression levels of torsinAΔE, an ER-resident protein, constitute an interesting group of factors that may unveil new aspects of yeast cell biology and of the secretory pathway.

Yeast is a powerful system in which to perform genetic screens, not only due to its low cost, but most importantly because of the translatability into mammalian systems. Sixty percent of yeast genes are homologous to human genes, and the use of yeast as a model organism to study mammalian cell biology in health and disease has uncovered invaluable information with high therapeutic relevance (Karathia et al., 2011; Botstein and Fink, 2011). In fact, conserved factors involved in amyotrophic lateral sclerosis, mitochondrial disorders, α-antitrypsin deficiency, prion diseases, CDGs, cystic fibrosis, kidney diseases, cancer, and Alzheimer's, Parkinson's, and Huntington's diseases, among many others, have been identified using yeast (Walberg, 2000; Winderickx et al., 2008; Bharadwaj et al., 2010; Kryndushkin and Shewmaker, 2011; Sarto-Jackson and Tomaska, 2016; Willingham et al., 2003; Giorgini et al., 2005; Youker et al., 2004). Given the past success of our yeast system in identifying relevant factors involved in torsinA and torsinAΔE biogenesis (Zacchi et al., 2014), and the demonstrated efficacy of yeast as a model organism, we anticipate that many of the genes identified in our screen will prove relevant in mammalian cells. Moreover, an advantage of unbiased genetic screens over targeted studies is that the identified hits provide innovative views of disease mechanisms that would have otherwise remained undiscovered. This is the first genome-wide screen to identify modifiers of the biogenesis and degradation of the EOTD-associated variant torsinAΔE. Together with the hits from a recent screen for effectors of torsinAΔE ER/NE subcellular localization (Rittiner et al., 2016), our results may enrich the pool of disease modifiers for EOTD with therapeutic potential. Beyond their potential role as modifiers of torsinA/ΔE expression levels, the hits identified here may help increase understanding of the complex biological machinery that plays a key role in maintaining protein homeostasis, potentially uncovering novel and unexpected players, pathways and interactions with broad impact in cell physiology. Finally, the new screening method we described here can be adapted to the study of other proteins of interest, providing insights on the biogenesis of any protein, either native to yeast or through heterologous expression.

## MATERIALS AND METHODS

### Plasmid construction

All vectors used in this study are described in Table 3. pRS425-torsinAΔE-HA was constructed by subcloning torsinAΔE-HA, including the *GPD* promoter and *CYC1* terminator, from pRS426-torsinAΔE-HA (Zacchi et al., 2014) by *Xho*I/*Bam*HI double digestion into pRS425. All cloned material was fully sequenced to ensure no mutations were introduced.

**Table 3.**
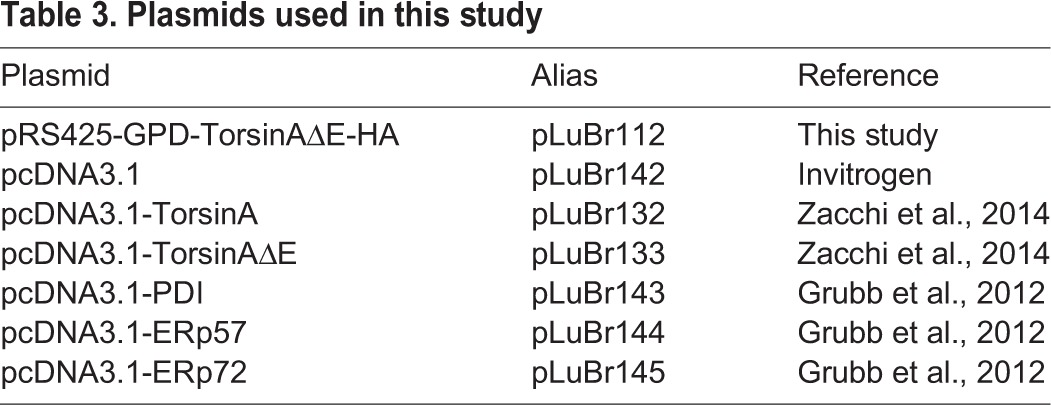
**Plasmids used in this study**

### Yeast strains, media and growth conditions

All yeast strains used are described in Table 1, and were grown at 28-30°C on YPD medium (1% yeast extract, 2% peptone, 2% dextrose) or on synthetic complete (SC) medium lacking specific amino acids required for auxotrophic selection, as previously described (Zacchi et al., 2014). Media was supplemented with 1 g l^−1^ 5-FOA and/or 2% galactose instead of glucose, when indicated. Yeast transformations were performed using lithium acetate/PEG3350, following standard protocols (Becker and Lundblad, 2008).

### Genetic screen

To screen for genes involved in torsinAΔE expression, we performed a high-throughput yeast mating and yeast colony-dot-blot screen by merging two published methods (Reid et al., 2011; Gelling et al., 2012; McCracken et al., 1996), and optimized published data analyses scripts (Dittmar et al., 2010; Appendix S1) to measure dot-blot signal intensity (Fig. 1; Fig. S1; and see Results). Briefly, the pRS425-GPD-torsinAΔE-HA expression vector was transformed into a *MAT***a** universal donor strain (Reid et al., 2011) (strain W8164-2C) (Fig. 1, Table 1). An overnight culture of the transformed universal donor strain was replica pinned in quadruplicate onto YPD plates, and mated to ∼4300 strains corresponding to the non-essential *MAT*α yeast deletion collection library (Open Biosystems) (Fig. 1A) by replica pinning the library on top of the donor strain spots. In this way, independent quadruplicate colonies for each deletion strain were generated (Fig. 1B, the white square indicates a quadruplicate of one deletion strain, *rsb1*Δ). To control for faster colony growth on the plate periphery, we used a pre-arranged yeast library in which each of the 384-well plates contained 308 deletion strains surrounded by a border of a *his3*Δ ‘wild-type’ growth-buffer strain (Fig. S1A) (Reid et al., 2011). Cells were only allowed to mate for 6 h to prevent cross-over and meiosis, which may compromise the chromosomal content of the final strain (Reid et al., 2011). After the mating, the diploid colonies were replica-pinned on selective SC-LEU+5-FOA+galactose medium in which 5-FOA and galactose are used to counterselect for the donor strain chromosomes, and this process was repeated several times (Fig. 1) (Reid et al., 2011). Next, the entire collection of haploid, single-deletion mutants transformed with the pRS425-GPD-torsinAΔE-HA expression vector was replica-pinned onto BioTrace™ NT Nitrocellulose Transfer Membrane (PALL 66485) layered on selective medium and incubated for 8 h (Gelling et al., 2012) (Fig. 1C). Subsequently, colonies growing on top of the membranes were lysed *in situ* using lysis buffer (0.2 M NaOH, 0.1% SDS and 0.5% 2-mercaptoethanol) (Gelling et al., 2012; McCracken et al., 1996); yeast and residual material were rinsed from the membranes, and the membranes were prepared for western dot-blotting (Fig. 1C). All yeast crosses and replica-pinning were performed using a high-density 1536-pinning tool in quadruplicate format in an S&P Robotics workstation (BM3-BC) and a rectangular Thermo Scientific™ Nunc™ OmniTray™ (12-565-450).

### Immunoblotting and image analysis

Biochemical methods for cellular protein extraction and western blotting were previously described (Zacchi et al., 2014). The following antibodies were used for western blot analysis: horseradish peroxidase (HRP)-conjugated anti-HA, dilution 1:5000 (clone 3F10, Roche Applied Science) and mouse monoclonal anti-torsinA D-M2A8, dilution 1:1000 (Cell Signaling); mouse anti-PDI, dilution 1:2000 (ADI-SPA-891, Enzo); mouse anti-ERp57, dilution 1:2000 (ADI-SPA-725, Enzo); rabbit anti-ERp72, dilution 1:2000 (ADI-SPA-720, Enzo); rabbit anti-beta actin, dilution 1:5000 (ab8227, Abcam); and horse or goat HRP-conjugated anti-mouse or anti-rabbit IgG secondary antibodies, dilution 1:10,000 (Cell Signaling). Western blots were developed with Supersignal West Pico or Supersignal West Femto Chemiluminescent Substrate (Pierce) detection reagents and images were visualized using a Kodak 440CF Image Station, a Bio-Rad ChemiDoc XRS+ or an Amersham Imager 600 (GE Healthcare). The signal was quantified using ImageJ v1.46r (NIH, USA). A modified version of the ScreenMill software suite (Dittmar et al., 2010) that allows for quantification of normalized colony-blot signal (Fig. S1B, Table S1) was used to quantify the signal in ImageJ (Appendix S1). The background subtracted mode of CM Engine (Dittmar et al., 2010) was used to generate raw quantifications of the dot-blot images. Owing to the occasional uneven exposure of the dot blots, the quantifications needed to be normalized so that measurements in one area of a membrane would be comparable to measurements in another area. To perform this normalization, a virtual box was centered around each dot, encompassing two additional dots in all directions. The variance of dot intensities within this box was calculated and then the box was shifted by one row or column, and again the variance was calculated (Fig. S1). This process of shifting the box by one position was repeated until all possible combinations of arrangements were considered around the dot in question. The mean of colony measurements from the box with the lowest variance was then selected as the normalization value for the dot in question. Additional details regarding this normalization can be found in Appendix S1. Ultimately, the positive hits (*P*<0.1) were selected using this modified ScreenMill method and were confirmed/supplemented by visual inspection of the images (Fig. S1B, Tables S1 and S2).

The list of hits obtained (yeast genes) (Table S2) was transformed to UNIPROT IDs (http://www.uniprot.org/uploadlists/) to search for GO using DAVID v6.8 (https://david.ncifcrf.gov/) (Table S3, GO). GO analyses were also performed using the GO-term Slim Mapper available at the SGD website (http://www.yeastgenome.org/) (Table S3, Component, Function, Process). Human homologs corresponding to the yeast hits were identified using Yeast Mine (http://yeastmine.yeastgenome.org) and BioMart (http://www.ensembl.org/biomart) (Table S4). Human Mine (www.humanmine.org/) (Smith et al., 2012) was used to identify human genes associated with disease (Table 2; Table S5). The Human Protein Atlas v16.1 was used to determine which genes were expressed in the brain (www.proteinatlas.org) (Uhlen et al., 2015) (Table S5, in bold).

### TorsinA and torsinAΔE expression in HeLa cells

HeLa cells (ATCC, USA) were maintained in DMEM (Gibco) supplemented with 10% fetal bovine serum at 37°C in a 5% CO_2_ humidified incubator. Cell lines were routinely checked for microbial contamination. Co-transfection of expression vectors for torsinA and the indicated human PDIs or the empty vector (Table 3) was performed by transfecting a total of 0.5 µg of vector (0.25 µg of each vector) using Lipofectamine 2000 or 3000 (Invitrogen) following the manufacturer's instructions. The medium was changed ∼4.5-5 h post-transfection. Protein extracts were prepared from cells harvested 24 h after vector transfection, as previously described (Zacchi et al., 2014).

### Statistical analysis

The normalized data from the screen approximated a normal distribution and therefore traditional statistical methods were used to derive *P*-values. Specifically, the mean and standard deviation of the distribution were calculated and from these values *z*-scores were derived. From the *z*-scores, two-tailed *P*-values were calculated by multiplying by two the value returned by sending the value of a *z*-score into the ‘uprob’ function of the Perl Statistical Distributions module (http://search.cpan.org/perldoc?Statistics::Distributions); *P*<0.1 was considered significant. Statistical analyses of the SDS-PAGE western blot data were performed using Student's *t*-test (Microsoft Excel Software), assuming unequal variances; *P*<0.05 was considered significant.
